# Disturbed retrieval network and prospective memory decline in postpartum women

**DOI:** 10.1038/s41598-018-23875-5

**Published:** 2018-04-03

**Authors:** Na-Young Shin, Yunjin Bak, Yoonjin Nah, Sanghoon Han, Dong Joon Kim, Se Joo Kim, Jong Eun Lee, Sang-Guk Lee, Seung-Koo Lee

**Affiliations:** 1 Department of Radiology, College of Medicine, The Catholic University of Korea, Seoul, Korea; 2 Department of Psychology, Yonsei University, Seoul, Korea; 3 Department of Radiology, Yonsei University College of Medicine, Seoul, Korea; 4 Department of Psychiatry, Yonsei University College of Medicine, Seoul, Korea; 5 Department of Anatomy, Yonsei University College of Medicine, Seoul, Korea; 6 Department of Laboratory Medicine, Yonsei University College of Medicine, Seoul, Korea

## Abstract

Prospective memory (PM) refers to the ability to remember to execute an intended action in the future. For successful PM performance, both top-down strategic monitoring and bottom-up spontaneous retrieval processes need to be appropriately recruited. We assessed PM performance and used fMRI to discover relevant neural correlates and possible predictors for PM performance in 25 postpartum and 26 nulliparous age- and education-matched women. Postpartum women showed decreased PM performance, a higher number of nocturnal awakenings, and lower estradiol level. The postpartum women had decreased functional connectivity (FC) in the right hippocampus and ventral frontoparietal networks (FPN) during retrieval-dominant PM trials relative to maintenance-dominant ongoing trials in the PM block. On multivariate analyses, decreased FC between the right hippocampus and ventral FPN and a higher number of nocturnal awakenings were independent predictors for poor PM performance after adjusting for age, education, estradiol level, and depressive symptoms. On mediation analyses, the estradiol level was found to have an indirect effect on PM accuracy via altered FC as a mediator. This suggests that decreased FC within the spontaneous retrieval-related regions including the right hippocampus and ventral FPN, disrupted sleep rhythms, and decreased estradiol level may contribute to poor PM performance in postpartum women.

## Introduction

More than half of pregnant women perceive a decline in their cognitive abilities after pregnancy^1^. Postpartum women also complain of similar, but maybe a milder degree of cognitive change^2^. “Maternal amnesia”, “momnesia” or “pregnancy brain” are colloquial terms for this condition and the most frequent symptoms are forgetfulness and memory disturbances^3,4^. Recent meta-analysis studies show that these symptoms are not just subjective, but objective cognitive impairments^2,5^.

Prospective memory (PM) refers to the ability to remember to execute delayed intention in appropriate situations^6^. PM requires planning and forming an intention, maintaining the memory of the intention, detecting cues and retrieving the related intention, and then executing the intention while performing an ongoing activity^7^. PM is essential when performing daily tasks, so disturbances to its processes can cause simple inconveniences or more major consequences. For instance, forgetting to attend an important meeting can lead to a missed promotion at work, forgetting to take medication can result in serious health problems, and forgetting to turn off the gas can even cause fires. Increased background cognitive demands^8^, depressive symptoms^9–11^, and sleep disturbance have been suggested as factors that negatively affect PM^12^, while exposure to estrogen^13,14^ has been suggested to positively affect PM. Postpartum women might be especially vulnerable to PM dysfunction because they are dealing with additional demanding ongoing tasks (e.g., feeding their baby or changing their baby’s diaper every couple of hours without sleep), while possibly suffering from depressive symptoms^15^, and because they have decreased serum levels of estrogen. A few studies have reported decreased PM performance in pregnant or postpartum women using behavioral data^4,16^. However, as these studies mainly focused on pregnant women, there is little information about how PM is affected during the postpartum period. Moreover, no study has focused on revealing culprit regions related to PM performance in postpartum women using functional magnetic resonance imaging (fMRI). PM might be more crucial to postpartum women as they now have to be mothers on top of their other family life roles, and in cases when they have to return to their jobs, they must also be proficient at their work. So, we need to verify whether postpartum women truly have decreased PM, and if so, we need to define what brain regions and clinical factors are associated with this decreased PM ability to develop more strategies to improve PM performance.

Therefore, the present study aimed to assess PM performance in postpartum women by comparing PM performance between postpartum women and nulligravid women. PM task-based fMRI was used to find neural substrates associated with PM performance in postpartum women. We also obtained clinical information to establish possible predictors for PM performance in postpartum women.

## Results

### Participant characteristics

Initially, 25 women were recruited for the Postpartum group and 30 women were recruited for the Control group. From the Control group, one participant was excluded due to excessive imaging artifacts, two due to movements exceeding a prior maximum movement of 2 mm, and one due to an incidentally found old infarct. No participants were excluded from the Postpartum group. Consequently, 25 participants in the Postpartum group and 26 in the Control group were included for final analysis. The demographic and clinical data of the participants are summarized in Table 1. All of the Postpartum group were married, while only 23.1% of the Control group were married (*P* < 0.001). Compared with the Control group, the Postpartum group complained of severer subjective cognitive decline (higher Cognitive Failure Questionnaire [CFQ] scores; *P* = 0.021). Although the Postpartum group tended to sleep longer per day (*P* = 0.084), they awakened more frequently during the night (*P* < 0.001) than the Control group and no difference was found in the self-rated scale for lack of sleep. The degree of depressive symptoms tended to be severer (higher Beck Depression Inventory [BDI] score) in the Postpartum group (*P* = 0.092). The serum estradiol level (*P* = 0.004) was significantly lower in the Postpartum group. Among the cognitive assessment tests, the Postpartum group exhibited poorer performance only in the digit span sequencing test compared with the Control group (*P* = 0.033). After the false discovery rate (FDR) correction, significant differences were observed for marital status, number of nocturnal awakenings, and serum estradiol level between the Postpartum group and the Control group. Within the Control group, age was the only factor to differ according to marital status, with single women being younger than married women (28.7 ± 3.7 years vs. 33.5 ± 2.4 years; *P* = 0.006). Within the Postpartum group, compared to women who did not breastfeed, breastfeeding women awakened more frequently during the night (2.0 [1.0–2.3] vs. 0.0 [0.0–2.0]; *P* = 0.021) and complained of severer subjective cognitive decline (39.4 ± 13.5 vs. 29.2 ± 9.7; *P* = 0.046). However, the differences in each group were not significant after FDR correction.

**Table 1:** Demographic and clinical characteristics of the participants.

	Control group (n = 26)	Postpartum group (n = 25)	*P* value
Demographic characteristics			
Age (y)	29.8 ± 4.0	30.9 ± 3.0	0.267
Duration of education (y)	19 (16–19)	16 (16–19)	0.160
Married	6 (23.1%)	25 (100%)	<0.001*
Normal delivery/C-sec	N/A	17/8	N/A
Interval between MRI scan and delivery date (d)	N/A	103.7 ± 16.2	N/A
Breastfeeding (at night)	N/A	14 (11)	N/A
Self-report questionnaires			
Number of awakenings	0.0 (0.0–0.0)	1.5 (0.0–2.0)	<0.001*
Total sleep time per day (h)	6.0 (6.0–6.8)	6.8 (6.0–8.0)	0.084
Lack of sleep	4.5 (2.0–7.0)	5.0 (3.0–6.0)	0.681
EPDS	6.1 ± 4.0	7.3 ± 5.6	0.446
BDI	6.2 ± 5.6	9.0 ± 6.0	0.092
CFQ	27.5 ± 9.3	34.9 ± 12.9	0.021
Estradiol assay (pg/mL)	87.0 (52.0–201.0)	40.0 (24.8–65.5)	0.004*
Neuropsychological tests			
COWAT (Animal)	21.0 ± 4.0	20.5 ± 5.8	0.725
15-item K-BNT	14.5 (14.0–15.0)	14.5 (14.0–15.0)	0.573
Digit span forward	15.0 (14.0–16.0)	14.0 (13.0–15.0)	0.127
Digit span backward	12.4 ± 2.8	11.6 ± 2.8	0.269
Digit span sequencing	9.5 ± 2.2	8.0 ± 2.5	0.033
Word List Memory	25.9 ± 2.9	24.9 ± 3.6	0.281
Word List Recall	90.0 (89.0–100.0)	100.0 (90.0–100.0)	0.141
Word List Recognition	10.0 (10.0–10.0)	10.0 (10.0–10.0)	0.579
TMT_A	20.0 (19.0–23.0)	20.0 (17.8–28.0)	0.962
TMT_B	62.3 ± 26.7	54.1 ± 22.4	0.242

*Found to be significant after false discovery rate correction for multiple comparisons. Abbreviations: BDI, Beck Depression Inventory; CFQ, Cognitive Failure Questionnaire; COWAT, Controlled Oral Word Association test; EPDS, Edinburgh Postnatal Depression Scale; 15-item K-BNT, 15-item Korean version of the modified Boston Naming Test; and TMT, Trail Making Test. Data that have normal distribution are expressed as mean ± standard deviation; otherwise, data are expressed as medians with interquartile ranges in parentheses.

### PM task behavioral performance

All participants successfully performed PM tasks for nearly all the trials, as measured by the proportion of correct responses (0.95 ± 0.05). The summary of correct responses and errors for the PM task are presented in Table 2. Compared to the Control group, the Postpartum group showed poorer performance in tasks requiring delayed intention. The Postpartum group gave fewer correct answers (*P* = 0.003) and had more omission errors (*P* = 0.003) with longer reaction times (*P* = 0.003) in PM trials, and fewer correct answers (*P* = 0.033) and more wrong errors (*P* = 0.033) in PM ongoing trials. Although, there were no significant differences in any of the performances for control (CTRL) ongoing trials, the Postpartum group tended to need more time to perform CTRL ongoing trials (*P* = 0.063). PM performance did not differ according to marital status within the Control group, nor did it differ according to breastfeeding within the Postpartum group.

**Table 2:** Correct responses and errors in the PM task.

	PM Block						CTRL Block		
	PM trial			PM ongoing trial			CTRL ongoing trial		
	Control	PP	*P* value	Control	PP	*P* value	Control	PP	*P* value
Correct response	31.0 (30.0–32.0)	29.0 (26.8–31.0)	0.003	32.0 (31.0–32.0)	31.0 (29.0–31.3)	0.033	63.0 (62.0–64.0)	62.0 (61.0–64.0)	0.107
Type of error									
Wrong	—	—	—	0.0 (0.0–1.0)	1.0 (0.8–3.0)	0.033	1.0 (0.0–2.0)	2.0 (0.0–3.0)	0.107
Omission	1.0 (0.0–0.2)	3.0 (1.0–5.3)	0.003	—	—	—	—	—	—
Commission	—	—	—	0.0 (0.0–0.0)	0.0 (0.0–1.0)	0.361	—	—	—
Reaction time (ms)	1248.0 ± 250.4	1460.7 ± 243.9	0.003	1829.8 ± 332.4	1948.3 ± 278.9	0.175	1132.8 ± 220.6	1248.6 ± 213.2	0.063

Abbreviations: PP, postpartum group. Data that have normal distribution are expressed as mean ± standard deviation; otherwise, data are expressed as medians with interquartile range in parentheses.

### PM task-based fMRI Results

#### PM task-related activation analysis

**Figure 1:**
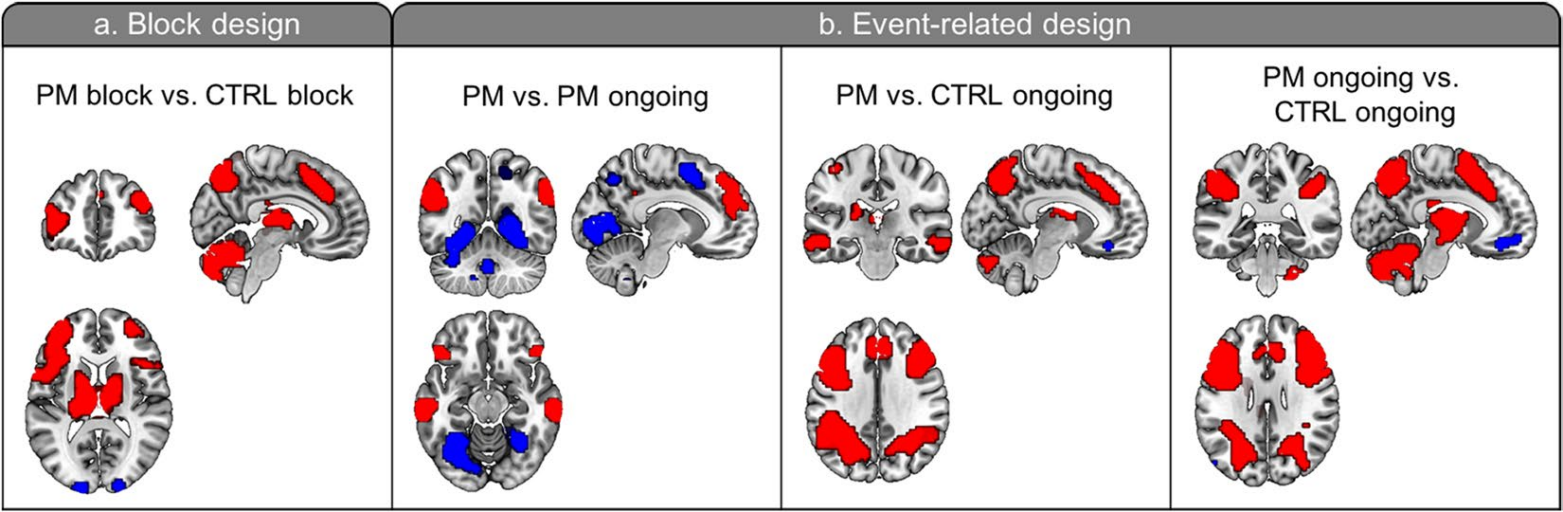
Main effect of tasks across all participants. (**a**) Block design analysis. Red clusters indicate increased activation, while blue clusters indicate decreased activation in the PM block compared to the CTRL block. (**b**) Event-related design analysis. Red clusters indicate increased activation, while blue clusters indicate decreased activation in PM trials compared to PM ongoing trials, PM trials compared to CTRL ongoing trials, and PM ongoing trials compared to CTRL ongoing trials, respectively. Images are oriented according to neurological convention (right is right; FWE-corrected *P* < 0.05).

In block and event-related design analyses, clusters including the bilateral rostrolateral prefrontal cortex (rlPFC), superior medial frontal gyrus, and lateral parietal cortex, areas which have been known to be associated with PM^17^, were found for the main effect of “task” (FWE-corrected *P* < 0.05; Fig. 1 and Supporting Information Table 1).

**Figure 2:**
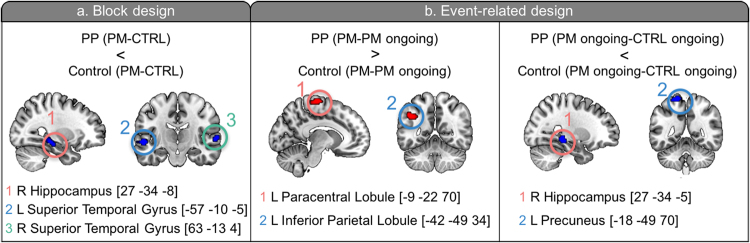
Clusters which had significant interaction of group x task on block design analysis (**a**) and event-related design analysis (**b**). PP, Postpartum group. Images are oriented according to neurological convention (right is right).

In the block design analysis, the right hippocampus and bilateral superior temporal gyri showed significant group x task interaction. In the event-related analysis, four clusters were found for the group x task interactions. The Postpartum group showed less activation increase in the right hippocampus and left precuneus in PM ongoing trials compared to CTRL ongoing trials. In contrast, the Postpartum group demonstrated greater activation increase in the left inferior parietal lobule (IPL) and right paracentral lobule in PM trials compared to PM ongoing trials (Fig. 2 and Supporting Information Table 2).

#### PM task-related FC analysis

**Figure 3:**
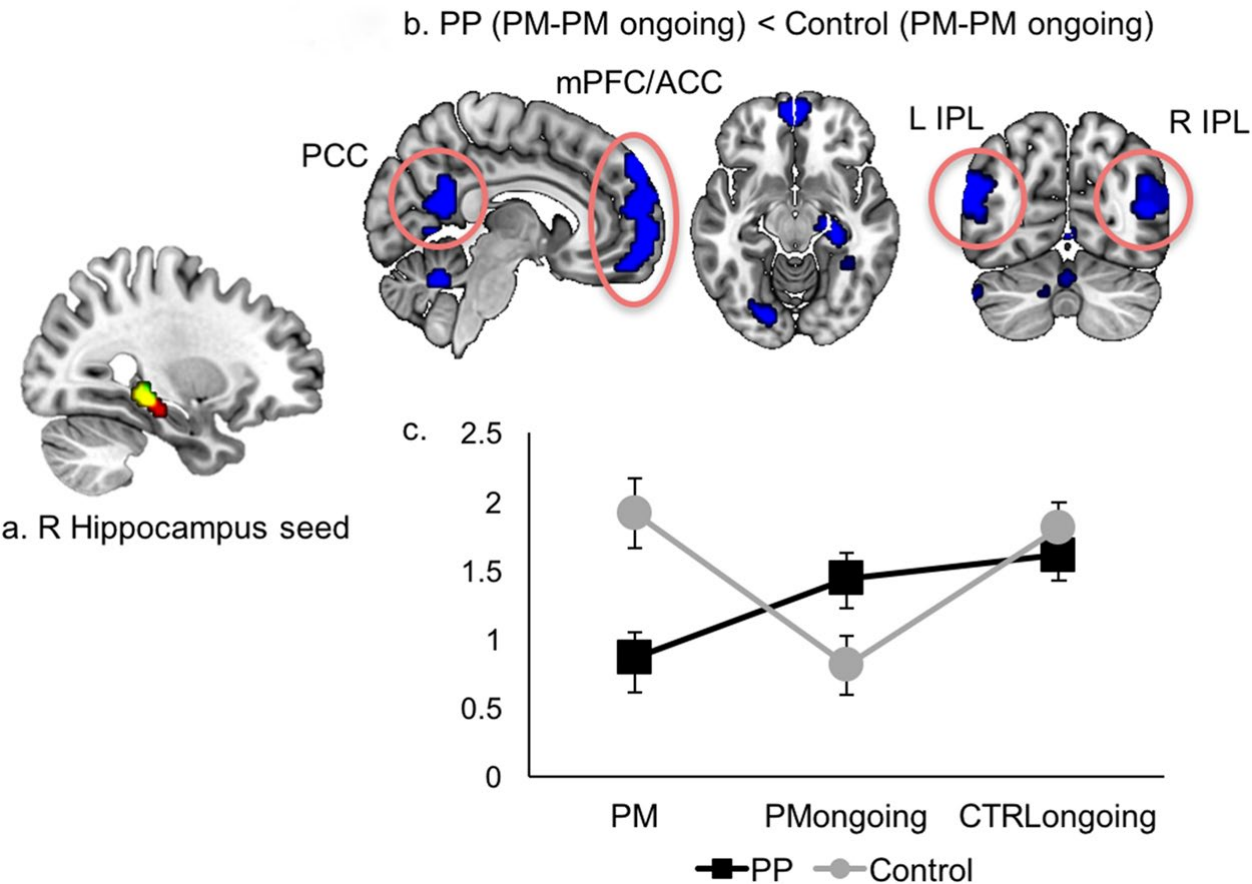
PM task-based FC analysis using the right hippocampus seed. (**a**) Right hippocampus seed (yellow cluster) which is a conjunction overlapping area between significant clusters with group x task interactions on block- (red cluster) and event- related (green cluster) design analyses. (**b**) Clusters showing less increase in FC in PM trials relative to PM ongoing trials in the Postpartum group. (**c**) Changes in the mean of beta estimates extracted from a mask comprised of 4 spherical ROIs (4 mm diameter each) centered at the peaks of the four main clusters (circles in (**b**) right and left inferior parietal lobules, medial prefrontal cortex/anterior cingulate cortex, and posterior cingulate cortex) according to the trial. ROI, region of interest.

**Figure 4:**
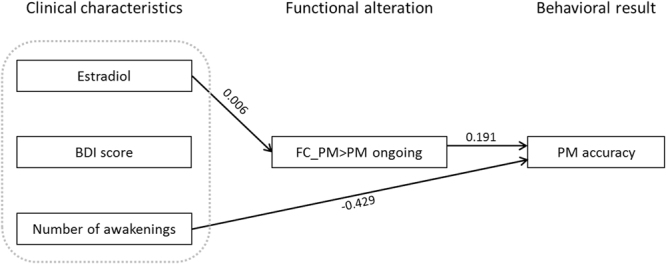
Schematic diagram of mediation analyses for PM accuracy. Serum level of estradiol, BDI score, and number of nocturnal awakenings were entered as predictors. FC_PM > PM ongoing (FC between the right hippocampus and retrieval-related ventral FPN in PM trials compared to PM ongoing trials) was entered as a mediator. Numbers on the paths are beta coefficients that were statistically significant after controlling for age and duration of education.

As the right hippocampal clusters were found to be significant in group x task interactions on both block and event-related design analyses, this region was thought to play a critical role in the decreased PM performance observed in the Postpartum group. So, we conducted a conjunction analysis with two clusters and selected the overlapping region as a seed for a generalized Psycho-Physiological Interaction (gPPI) analysis (Fig. 3a). Moreover, meta-analysis results using the Neurosynth framework (http://neurosynth.org/) which is a platform for automated meta-analysis and available via a web interface showed that this region was selectively related with PM, supporting our assumption. The Postpartum group revealed less increase in FC in the bilateral IPL, posterior cingulate cortex (PCC), and medial prefrontal cortex (mPFC)/anterior cingulate cortex (ACC) in PM trials relative to PM ongoing trials (Fig. 3b and Supporting Information Table 3), which substantially overlapped with the retrieval-related ventral frontoparietal network (FPN) discussed in a recent meta-analysis study^18^. We extracted beta estimates, representing the strength of FC with the hippocampus seed, from the four main clusters (PCC, mPFC/ACC, right and left IPLs) included in this network, and revealed that the interaction was based on a significant decrease in FC in PM trials compared with PM ongoing trials in the Postpartum group (*P* < 0.001), while there was a significant increase in FC in PM trials compared with PM ongoing trials in the Control group (*P* = 0.005; Fig. 4c). In PM trials relative to CTRL ongoing trials, the Postpartum group showed less increase in FC mainly in the occipital and temporal areas. However, in PM ongoing trials relative to CTRL ongoing trials, the Postpartum group showed an increase in FC in the ventromedial PFC (Supporting Information Table 3).

### Clinical and imaging variables associated with PM accuracy

Decreased PM accuracy was correlated with a higher BDI score (greater depressive symptoms, *ρ* = −0.320, *P* = 0.024), a higher number of nocturnal awakenings (*ρ* = −0.393, *P* = 0.005), a lower FC between the right hippocampus and retrieval-related ventral FPN in PM trials compared to PM ongoing trials (*ρ* = 0.306, *P* = 0.029), a lower score on the Digit span sequencing test (*ρ* = 0.322, *P* = 0.021), and a higher CFQ score (greater subjective cognitive decline; *ρ* = −0.268, *P* = 0.057). On the generalized linear model analysis, the number of nocturnal awakenings (odds ratio [OR] = 0.651 [0.525–0.809]; *P* < 0.001) and FC between the right hippocampus and ventral FPN in PM trials relative to PM ongoing trials (OR = 1.211 [1.034–1.419]; *P* = 0.018) were independent predictors for PM accuracy even after adjusting for age, duration of education, serum estradiol level, and the BDI score (Table 3). The mediation analyses for PM accuracy showed that a decreased serum level of estradiol was associated with decreased FC between the right hippocampus and ventral FPN in PM trials relative to PM ongoing trials that led to decreased PM accuracy. An increased number of nocturnal awakenings was associated with decreased PM accuracy without being mediated by altered FC. The BDI score did not significantly affect PM accuracy in both direct or indirect ways (Fig. 4 and Supporting Information Table 4).

**Table 3:** Multivariate analyses of factors predicting PM accuracy.

Variable	Odds ratio	95% CI	*P* value
Age (y)	1.039	0.981–1.102	0.192
Duration of education (y)	1.000	0.900–1.111	0.965
Estradiol (pg/mL)	1.001	0.998–1.004	0.444
BDI score	1.036	0.994–1.080	0.094
Number of awakenings	0.651	0.525–0.809	<0.001
FC_PM > PM ongoing	1.211	1.034–1.419	0.018

Abbreviations: BDI, Beck Depression Inventory; CI, confidence interval; FC_PM > PM ongoing, functional connectivity between the right hippocampus and retrieval-related ventral fronto-parietal network in PM trials compared to PM ongoing trials.

## Discussion

Our study revealed decreased PM performance and altered PM task-related brain activity in the Postpartum group. The right hippocampus failed to show significant increase in PM-related brain activation and had decreased FC with the ventral FPN in PM trials relative to PM ongoing trials in the Postpartum group. Furthermore, along with the number of nocturnal awakenings, FC between the right hippocampus and ventral FPN in PM trials relative to PM ongoing trials, which mediates the effect of the serum level of estradiol, was an independent predictor of PM performance. These findings suggest that the right hippocampus and its connectivity with the ventral FPN might be the culprit neural correlates for decreased PM performance in postpartum women.

According to the multiprocess framework, largely two kinds of processes may support PM: top-down strategic monitoring and bottom-up spontaneous retrieval processes^19^. Top-down strategic monitoring refers to the sustained attentional control process that maintains an intention active in the mind while a person is performing other ongoing tasks^20^ and that monitors the environment for intention relevant stimuli^21^. rlPFC and dorsal FPN (i.e., dorsolateral PFC, precuneus, and superior parietal lobule) have been suggested to subserve these processes^17,18,22^. Sometimes, the retrieval of PM intention is spontaneously and automatically triggered by certain PM cues without any efforts to keep the PM intention active. This is referred to as the bottom-up spontaneous retrieval process^23^. According to a recent meta-analysis study^18^, the ventral FPN (i.e., ventrolateral PFC, ACC, PCC, IPL, and insula) and ventromedial aspects of rlPFC might be neural correlates for this process.

In our study, we found that postpartum women had altered activation in the right hippocampus. Although inconclusive^24,25^, the medial temporal lobe including the hippocampus has also been suggested to be involved with the retrieval process, specifically in the more reflexive and automatic retrieval process recruited only by particular PM cues (i.e., focal PM cues, for which required cognitive processes are similar to those required during ongoing tasks)^24,26^. In the present study, we used focal PM cues (i.e., both PM and ongoing tasks required judgement on the direction of the hour hand of the clock), and the right hippocampus might have been consequentially recruited in the retrieval process, leading to group differences. Some studies have also reported that the right hippocampus, particularly its posterior portion as shown in our study, is associated with successful PM^27^ and future event construction^28^.

For successful PM performance, the regions relevant to each process must connect well at appropriate moments. Therefore, we conducted a gPPI analysis to evaluate PM-related FC patterns. Because intention retrieval is a more predominant process in the PM trial compared to the PM ongoing trial, FC between regions relevant to the retrieval process was expected to increase, as shown in the Control group. In contrast to the Control group, however, the Postpartum group showed decreased FC between retrieval-related regions in PM trials relative to PM ongoing trials, and this might be the reason behind poor PM performance in postpartum women. Supporting this assumption, FC between the right hippocampus and ventral FPN was an independent predictor for PM performance even after adjustment of possible confounding factors including age, duration of education, quality of sleep, serum estradiol level, and depressive symptoms.

Individuals rely on top-down strategic monitoring and bottom-up spontaneous retrieval processes to varying extents depending on the characteristics of PM and ongoing tasks as well as individual factors^19,29,30^. When the PM cue is focal and salient, it can be detected without much effort and when a person is performing very important and demanding ongoing tasks, there are not enough cognitive resources for sustained attentional control; thus, in these cases, individuals tend to depend more on the spontaneous retrieval process^19,30^. Given that postpartum women probably rely more on the spontaneous retrieval process for PM due to an increase in demanding ongoing tasks (e.g., taking care of their babies), disturbed neural correlates for the retrieval process might cause poor PM performance in their daily lives. Moreover, Hoekzema *et al*.^31^ have recently reported that postpartum women showed gray matter atrophy in the mPFC, precuneus, and bilateral temporal and frontal areas including the bilateral hippocampi which subserve social cognition and these areas show substantial overlap with the ventral FPN reported in our study. The authors also found more response in these regions when each woman was shown a picture of her own baby compared to when they were shown a picture of an unknown baby. Accordingly, the authors suggested that structural change might be an adaptive process for motherhood^31^. Therefore, we can also postulate that functional and structural alterations of the ventral FPN and hippocampus are critical changes for becoming a mother and PM cues, such as the unknown baby, might not be able to sufficiently stimulate these areas in postpartum women compared to signs of their own babies.

Although the serum estradiol level was not correlated with PM performance and was unable to predict PM performance in a direct way, it affected PM performance via altered FC during the PM task in our study.

This is consistent with previous results. It has been widely accepted that estrogen has neuroprotective effects on cognitive functions including working memory, verbal memory, and prospective memory in postmenopausal^14,32^ as well as premenopausal^33,34^ women. Estrogen has been suggested to be associated with cognitive function via modulation of brain activation^35^ and regional cerebral blood flow^13,36^ in particular regions including the inferior frontal gyrus, ACC, hippocampus, and IPL which overlap with the regions found in our study. Moreover, the presence of estrogen receptors in the hippocampus and cerebral cortex^37–39^ could be evidence of estrogen playing a role on cognition through brain functional modulation. Therefore, abrupt estrogen withdrawal after high serum levels of estrogen are sustained during pregnancy might contribute to decreased cognitive function including decreased PM in postpartum women. Unlike our results, some previous studies even found direct associations between estrogen and PM^13,14^. The use of different markers for estrogen (i.e., the Index for Cumulative Estrogen Exposure, which has been more consistently associated with cognition^40^, instead of the serum estrogen level measured at a single time point) and individual variability in the estrogen receptor gene which would result in different estrogen activity in the brain^41^ might be possible explanations for this discrepancy.

Several studies have demonstrated that individuals with sleep deprivation^42^ and bad sleeping habits^12^ have decreased PM performances. In line with previous results, a higher number of nocturnal awakenings was an independent predictor for poor PM performance and it affected PM performance without being mediated by altered FC during the PM task in our study. This finding is significant because sleep disturbance is a modifiable factor and appropriate interventions to improve quality of sleep might help postpartum women achieve better PM performance. It is worth noting that most breastfeeding women breastfed during the night (11/14, 78.6%) and had poorer sleep quality. Therefore, proper sleep education for this population could be the first step for better PM performance.

Depressive symptoms were also an important predictor of PM performance, although it failed to reach statistical significance. As we recruited only clinically non-depressed participants, the effects of depressive symptoms might have been underestimated.

Our study has several limitations. First, we only focused on event-based PM (i.e., retrieval of intention in response to an event) in this study as most neuroimaging research incorporates event-based PM tasks^17,22,43^, and there are already some hypotheses about the neurocognitive mechanism behind event-based PM^19,30,44,45^, which makes it easier to understand the significance of our results in relation to previous studies. Tasks for event-related PM are considered easier to perform than those for time-based PM (i.e. retrieval of intention after a defined amount of time which requires self-initiated processes)^46–48^, so our PM task was also easy to execute and most participants in our study were able to successfully perform the task for nearly all trials. Therefore, we could not evaluate brain activation patterns corresponding to error and correct trials, respectively. Second, we asked the study participants to only perform a PM task with focal PM cues and the attentional control process might not be required as much as the retrieval process for this task, causing false-negative findings. Therefore, studies using more cognitively demanding, non-focal or time-based PM tasks would be helpful to elucidate what brain regions are associated with error trials as well as the presence of altered top-down attention control-related brain activity during PM tasks in postpartum women. Finally, our cohort was not matched for marital status between the groups. However, we thought that marital status would not have a significant effect on PM as most studies researching the association between marital status and cognition have reported a positive long-term effect of marital status on mid- or later life cognition^49–51^. Also, there is little evidence of marital status having a relatively short-term effect on cognition including PM in young adults. Moreover, there was no difference in cognitive function according to marital status, including for PM accuracy within the Control group in our study. However, further studies with more well controlled cohorts are warranted for additional confirmative results.

In conclusion, the present study showed decreased PM behavioral performance in postpartum women compared to nulligravid control subjects. Decreased FC within spontaneous retrieval-related regions, serum estradiol level further affecting the altered FC, and disrupted sleep rhythms may contribute to poor PM performance in postpartum women.

## Methods

### Participants

Women aged 20–40 years in the 2^nd^–4^th^ month after parturition (Postpartum group) with normal pregnancies, uncomplicated term vaginal or Caesarean deliveries, and healthy babies were recruited. Women who were matched to these patients for age and duration of education and who had never been pregnant (Control group) were recruited for comparison. Participants were excluded if they had any history or comorbidities that might account for cognitive dysfunction. All participants were right-handed, native Koreans with normal or corrected-to-normal vision.

### Standard protocol approvals, registrations, and patient consents

This prospective study protocol was approved by the Yonsei University Severance Hospital ethical standards committee on human experimentation, and all participants provided written informed consent prior to all study procedures. All experiments were performed in accordance with relevant guidelines and regulations.

### Experimental tasks and procedures

Participants were asked to fill out self-report questionnaires for demographic and clinical information. Then, blood samples were drawn for the estradiol assay. After participants completed a shorter practice version of the fMRI scanning session using a laptop computer, PM task-based fMRI and a 3-dimensional (3D) T1-weighted image were acquired. Afterwards, a cognitive test battery was conducted to evaluate the objective cognitive status of the participants.

#### Self-report questionnaires

Self-report questionnaires were comprised of questions regarding demographic and clinical characteristics, total sleep time per day, number of nocturnal awakenings, and a scale of 10 to rate sleep deprivation, for which 0 = absence and 10 = very severe to indicate lack of sleep.

To assess depressive symptoms, both the Edinburgh Postnatal Depression Scale (EPDS)^52^ and the Beck Depression Inventory (BDI)^53^ were used, as EPDS, which was specifically developed and validated to measure depressive symptoms in postpartum women^54,55^, has not been validated for nulliparous women, while BDI, which is widely used to evaluate depressive symptoms, has not been validated for the detection of postpartum depression^56^. Moreover, previous studies have shown a strong correlation (*r* = 0.82) between EPDS and BDI scores^57^ and there were no significant differences in diagnostic performance for detecting postpartum depression when these two scales were used as continuous measures^58^; thus, we used both to quantify subjective depression for the Postpartum and Control groups while maintaining consistency between the two groups. Finally, the Cognitive Failure Questionnaire (CFQ)^59^ was included to assess subjective cognitive decline.

#### Estradiol assays

Blood samples were collected via venipuncture from the antecubital space and serum estradiol levels were measured by an enzyme chemiluminescent immunoassay using a UniCel DxI 800 automated analyzer (Beckman Coulter, Inc, Fullerton, CA). The minimum sensitivity and range of estradiol was 20–4800 pg/mL.

#### Neuropsychological tests

The cognitive test battery was comprised of the semantic Controlled Oral Word Association test (COWAT) and Trail Making Test (TMT) part B to assess executive function/verbal fluency^60^, the Digit Span subset of the Korean version of the Wechsler Adult Intelligence Scale: forward, backward, and sequencing recall tests to assess working memory^61^, the 15-item Korean version of the modified Boston Naming Test (K-BNT) to assess language function, the Word List Memory to assess verbal learning, the Word List Recall and Recognition to assess verbal learning/memory function, and the TMT part A to assess speed of information processing^60^.

#### fMRI paradigm for PM

**Figure 5:**
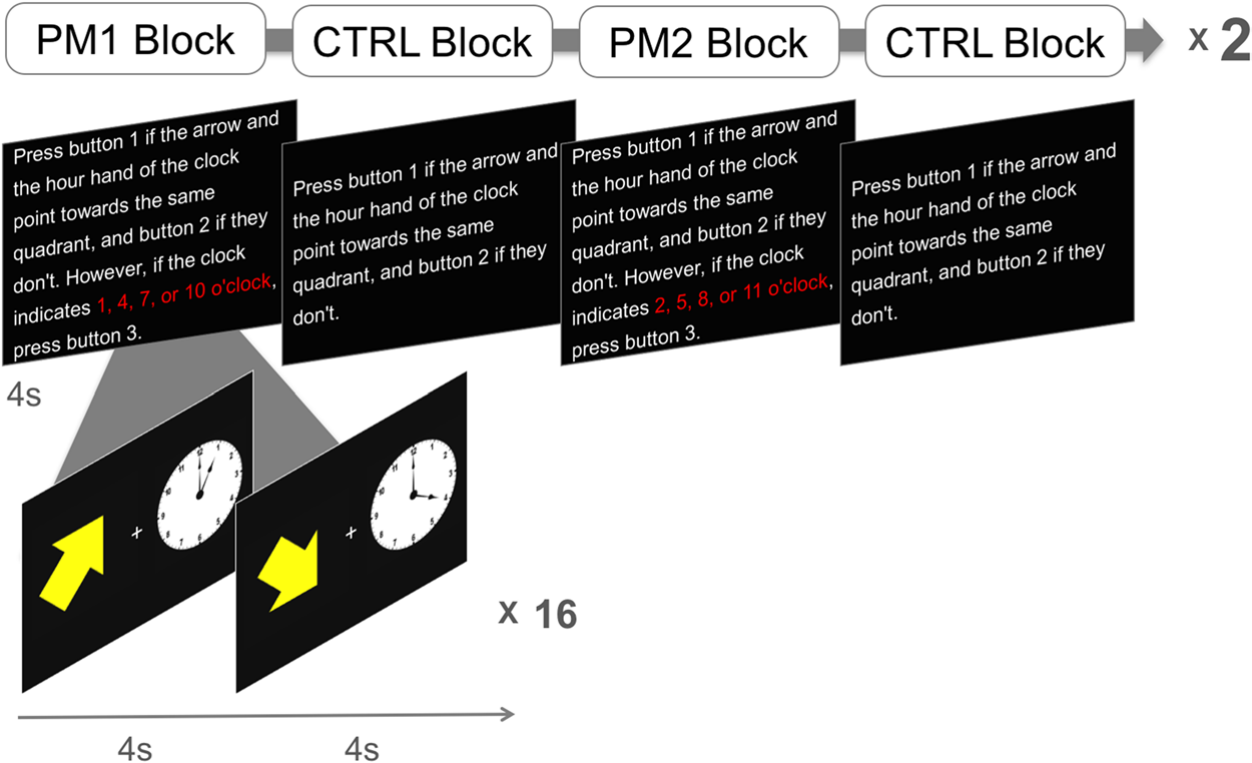
Schematic illustration of the PM task.

The PM task in our study has the typical features of a PM paradigm^43^ and was inspired by tasks used in previous studies^62,63^. The PM task was designed with alternating blocks of PM and CTRL conditions, and each block was repeated 4 times (Fig. 5). The stimuli consisted of an arrow and a clock presented in the left and right side of the screen, respectively. For the ongoing task, participants were instructed to respond by pressing a keypad with the right index or third finger depending on whether the arrow and the hour hand of the clock indicated the same quadrant or not. Both the arrow and the clock could indicate a direction from 1 to 11 o’clock. The CTRL blocks consisted of only ongoing trials (CTRL ongoing) where participants did not have to maintain PM intentions (uncontaminated ongoing trials; not affected by PM intention). In the PM blocks, participants were asked to remember particular times (1, 4, 7, 10 or 2, 5, 8, 11; each set was the target of 2 PM blocks), and to press a third button with their ring finger whenever they saw that particular time on the clock (PM trial) while performing the ongoing task (PM ongoing; contaminated ongoing trials; affected by PM intention). Each block consisted of 16 trials, and half of the trials were PM trials for the PM block. At the beginning of each block, an instruction page was presented for 4 s which gave directions on the tasks of that certain block. Participants were given 4 s to perform each following task trial. Responses were categorized into correct response, wrong error, omission error, and commission error. Correct response was defined as pressing the correct button in each trial, wrong error as failure to respond or pressing the wrong button in ongoing trials, omission error as failure to press the third button in PM trials, and commission error as failure to inhibit PM response in ongoing trials. The PM task was programmed using the Cogent 2000 toolbox (www.vislab.ucl.ac.uk/cogent.php) and MATLAB 7.12.0 (The MathWorks).

### Image acquisition

All scans were acquired by using a 3 T MR imaging unit (Discovery MR750; GE Healthcare, Milwaukee, WI) with an 8-channel head coil. fMRI data were acquired with a gradient-echo single-shot echoplanar imaging sequence with the following parameters: TR = 2000 ms, TE = 30 ms, FOV = 240 × 240 mm^2^, voxel size = 3.75 × 3.75 × 4.0 mm^3^, flip angle = 90°, 33 axial slices tilted 30° from the AC–PC plane, no gap, interleaved, scan time = 9 m 14 s including a dummy scan for 10 s. During each scan, stimuli were projected onto a black background screen at the end of the scanner, and participants viewed stimuli by way of a mirror mounted on the head coil. For coregistration and normalization purposes, we also performed a 3D-T1-turbo field echo sequence with the following parameters: sagittal acquisition with TR = 8.3 ms, TE = 3.3 ms, FOV = 198 × 220 mm^2^, voxel size = 0.77 × 0.86 × 1.0 mm^3^, 216 slices, flip angle = 12°, no gap.

### Image analysis

The fMRI data were preprocessed and analyzed using SPM8 (Wellcome Department of Cognitive Neurology, London, U.K.). Slice-timing correction was done by resampling all slices relative to the middle slice (i.e., 17^th^ slice) in temporal order. The EPI data for each subject was realigned to the first volume for motion correction and only data sets with ≤ 2 mm maximal displacement during the entire scan were included in this study. Next, the functional images were coregistered to the T1-weighted image and spatially normalized to the Montreal Neurological Institute (MNI) template provided with SPM8, then resampled into 3 × 3 × 3 mm voxels, followed by spatial smoothing using a Gaussian kernel with a full width at half maximum (FWHM) of 8 mm. A high-pass filter of 1/128 Hz was used to remove low-frequency noise, and an AR (1) + white noise model was used for temporal autocorrelation.

#### PM task-related activation analysis

General linear model analyses were then performed for both block and event-related designs to examine not only sustained but transient brain activity differences between the groups. In block design analysis, regressors were created for PM blocks and CTRL blocks by convolving the boxcar function with the hemodynamic response function (HRF) implemented in SPM. The length of each block in the regressors was 68 s which included 4 s to present the instruction page. In event-related analysis, regressors were created for each trial type by convolving neural input functions with the HRF. All error trials were separately modeled with a single regressor and excluded from further analyses. Six movement parameters extracted from the realignment process were included as confounders, as well as a single covariate representing the mean session effect. Random-effects group analyses were performed in full-factorial design with within-subject factors (block design: PM vs. CTRL block; event-related design: PM vs. PM ongoing vs. CTRL ongoing trial) and between-subject factors (group: Postpartum vs. Control) using beta estimates for each regressor.

#### PM task-related functional connectivity (FC) analysis

To identify group differences in PM task-related FC, a gPPI analysis^64^ was conducted. A right hippocampus seed was obtained from the overlapping area between significant clusters with group x task interaction from the block- and event-related design analyses. The physiological variable was made by deconvolving the mean BOLD signal within the seed, and the psychological variable was created by convolving each task regressor (i.e., PM, PM ongoing, and CTRL ongoing trials) with the HRF. The physiological variable was multiplied by the psychological regressors to form the interaction term. Individual beta images for each PPI regressor were then entered into group-level analysis with procedures identical to those used in event-related design analyses. To identify specific patterns of connectivity across conditions in regions which showed significant differences between the Postpartum and Control groups, ROI analyses were conducted using the MarsBar plug-in for SPM (http://marsbar.sourceforge.net/). The ROIs were converted to 4 mm sphere masks centered at the peak, and percent signal changes which were extracted from each ROI for each participant were entered into the second level analysis.

Unless stated otherwise, all statistical analyses were corrected for multiple comparisons based on Monte Carlo simulation corresponding to an alpha level of *P* < 0.05^65^.

### Clinical data analysis

The Kolmogorov-Smirnov test was used to determine whether data were normally distributed. Accordingly, quantitative data with normal distribution were presented as means ± standard deviations (SDs), and were compared by using a two-sample *t*-test. Otherwise, data were presented as medians with ranges and the Mann-Whitney *U*-test was used. Qualitative data were analyzed by using the χ^2^ test or Fisher’s exact test. False discovery rate correction was performed to correct for multiple comparisons in clinical variables and neuropsychological test results^66^. Two-tailed Spearman correlation analyses were performed to assess the relationship between PM accuracy and clinical and imaging results which either showed significant group differences or were suggested to be related to PM performance (e.g., depressive symptoms). We also conducted a univariate generalized linear model analysis to evaluate the association between PM accuracy and the significantly correlated variables in the above-mentioned Spearman correlation analysis. Then, a multivariate generalized linear model was used to identify independent factors for PM accuracy. Variables significantly associated with PM accuracy in the univariate model (*P* < 0.05), other variables of interest (e.g., serum level of estradiol and depressive symptoms), and possible confounding factors (e.g., age and duration of education) were included in the multivariate model. Additionally, to evaluate whether altered FC during the PM task mediated the effects of the clinical variables, mediation analyses were performed after controlling for age and duration of education using a generalized linear model.

Statistical analyses were performed by using SPSS, Version 24.0 (IBM, Armonk, New York), and a two-tailed *P* < 0.05 was considered significant.

### Data availability

The datasets analyzed during the current study are available from the corresponding author on reasonable request.

## Electronic supplementary material

Supplementary tables
