# Author Correction: Disturbed retrieval network and prospective memory decline in postpartum women

**DOI:** 10.1038/s41598-018-32260-1

**Published:** 2018-09-21

**Authors:** Na-Young Shin, Yunjin Bak, Yoonjin Nah, Sanghoon Han, Dong Joon Kim, Se Joo Kim, Jong Eun Lee, Sang-Guk Lee, Seung-Koo Lee

**Affiliations:** 1 Department of Radiology, College of Medicine, The Catholic University of Korea, Seoul, Korea; 2 Department of Psychology, Yonsei University, Seoul, Korea; 3 Department of Radiology, Yonsei University College of Medicine, Seoul, Korea; 4 Department of Psychiatry, Yonsei University College of Medicine, Seoul, Korea; 5 Department of Anatomy, Yonsei University College of Medicine, Seoul, Korea; 6 Department of Laboratory Medicine, Yonsei University College of Medicine, Seoul, Korea

Correction to: *Scientific Reports* 10.1038/s41598-018-23875-5, published online 03 April 2018

The Acknowledgements section in this article is incomplete:

“We would like to thank Kyunghwa Han for her statistical advice. This study was supported by the National Research Foundation of Korea Grant funded by the Korean Government (NRF-2014R1A1A2055116) and faculty research grants from Yonsei University College of Medicine (4-2013-0111).”

should read:

“We would like to thank Kyunghwa Han for her statistical advice. This research was supported by Basic Science Research Program through the National Research Foundation of Korea (NRF) funded by the Ministry of Education (2014R1A1A2055116) and faculty research grants from Yonsei University College of Medicine (6-2013-0058).”

