# Supplementary material for: Disturbed retrieval network and prospective memory decline in postpartum women
Source: Sci Rep. 2018 Apr 3;8:5476. doi: 10.1038/s41598-018-23875-5 (PMC5882973; doi:10.1038/s41598-018-23875-5)
Supplement: Supplementary file 1 — Supplementary tables [file 41598_2018_23875_MOESM1_ESM.pdf]

Title:

Disturbed retrieval network and prospective memory decline in postpartum women

Authors:

Na-Young Shin, MD, PhD[1], Yunjin Bak, MA[1], Yoonjin Nah, MA[2], Sanghoon Han PhD[2], Dong Joon Kim, MD, PhD[3], Se Joo Kim, MD, PhD[4], Jong Eun Lee, PhD[5], Sang-Guk Lee, MD[6], Seung-Koo Lee MD, PhD[3*]

Affiliations:

[1]Department of Radiology, College of Medicine, The Catholic University of Korea, Seoul, Korea

[2]Department of Psychology, Yonsei University, Seoul, Korea

[3]Department of Radiology, Yonsei University College of Medicine, Seoul, Korea

[4]Department of Psychiatry, Yonsei University College of Medicine, Seoul, Korea

[5]Department of Anatomy, Yonsei University College of Medicine, Seoul, Korea

[6]Department of Laboratory Medicine, Yonsei University College of Medicine, Seoul, Korea

Correspondence:

Seung-Koo Lee, MD, PhD

Department of Radiology, Yonsei University College of Medicine

50-1 Yonsei-ro, Seodaemun-gu, Seoul 120-752, Korea

Phone: (+82) 2-2227-2373   Fax: (+82) 2-393-3035

E-mail: slee@yuhs.ac

Supplementary Table 1. Significant regions for the main effect of the task

Design	Contrast	Region	Side	MNI coordinates			Maximum *t*	No. of voxels	*P* value
				x	y	z			
Block	PM block > CTRL block	Inferior parietal lobule	left	-33	-55	37	11.06	3341	< .001
		Frontal inferior operculum	left	-48	8	28	9.37	3425	< .001
		Supplementary motor area/superior medial frontal gyrus	left	-3	17	49	8.46	555	< .001
		Frontal inferior operculum	right	42	32	28	8.12	1208	< .001
		Cerebellum	right	9	-73	-32	7.55	2217	< .001
		Inferior temporal gyrus	left	-45	-55	-11	5.33	35	< .001
		Cerebellum	left	-21	-40	-44	4.97	6	< .001
	PM block < CTRL block	Superior occipital gyrus	right	18	-100	16	6.62	75	< .001
		Middle occipital gyrus	left	-15	-100	13	5.88	44	< .001
Event-related	PM > PM ongoing	Middle temporal gyrus	left	-54	-58	34	8.74	362	< .001
		Angular gyrus	right	57	-55	37	7.21	165	< .001
		Middle temporal gyrus	left	-63	-25	-11	7	200	< .001
		Middle temporal gyrus	right	69	-31	-11	6.94	155	< .001
		Postcentral gyrus	left	-45	-28	61	6.58	86	< .001
		Superiomedial frontal gyrus	left	-6	47	25	6.21	393	< .001
		Inferior temporal gyrus	left	-54	-1	-35	6.06	55	< .001
		Orbitoinferior frontal gyrus	right	45	29	-14	5.38	36	< .001
		Orbitoinferior frontal gyrus	left	-48	32	-14	5.31	62	< .001
		Inferior temporal gyrus	right	54	-4	-35	4.91	6	< .001
	PM < PM ongoing	Supplementary motor area	left	-24	-4	52	9.1	672	< .001
		Middle frontal gyrus	right	24	-1	49	8.8	271	< .001
		Lingual gyrus	right	27	-55	-8	7.07	1616	< .001
		Superior parietal gyrus	right	18	-70	46	6.18	155	< .001
		Vermis		0	-55	-38	6.13	67	< .001
		Insula	right	33	20	7	5.88	121	< .001
		Precentral gyrus	left	-57	8	28	5.84	95	< .001
		Superior parietal gyrus	left	-15	-67	46	5.75	75	< .001
		Putamen	left	-21	8	1	5.55	34	< .001
		Inferior frontal operculum	right	45	8	28	5.41	24	< .001
		Supramarginal gyrus	right	39	-40	43	5.33	49	< .001
		Cerebellum	left	-15	-55	-50	5.03	7	< .001
		Middle frontal gyrus	left	-39	59	16	4.98	10	< .001
		Inferior parietal gyrus	left	-42	-40	43	4.97	17	< .001
		Inferior frontal triangularis	left	-51	29	25	4.73	9	< .001
	PM > CTRL ongoing	Inferior parietal gyrus	left	-33	-58	40	10.33	2861	< .001
		Inferior frontal operculum	right	42	29	31	7.54	714	< .001
		Orbitomedial frontal gyrus	left	-45	20	28	7.49	2147	< .001
		Superior medial frontal gyrus		0	38	34	7.46	470	< .001
		Cerebellum	right	9	-76	-32	6.83	771	< .001
		Inferior temporal gyrus	left	-63	-25	-14	5.94	170	< .001
		Orbitomiddle frontal	right	48	38	-20	5.7	38	< .001

	gyrus							
	Inferior temporal gyrus	right	63	-22	-17	5.7	131	< .001
	Caudate	right	21	-1	22	5.36	81	< .001
	Cerebellum	left	-39	-67	-35	5.35	66	< .001
	Inferior temporal gyrus	left	-51	-49	-14	5.26	21	< .001
	Insula	right	33	23	-5	5.14	22	< .001
	Insula	left	-33	23	-5	5.06	13	< .001
	Middle frontal gyrus	right	30	56	13	5.05	14	< .001
	Inferior frontal triangularis	right	54	20	-2	4.87	27	< .001
	Vermis	right	3	-58	-29	4.84	9	< .001
	Vermis		0	-61	-17	4.7	2	< .001
	Inferior occipital gyrus	left	-33	-91	-11	4.63	2	< .001
	Supramarginal gyrus	left	-60	-22	19	4.6	3	< .001
PM < CTRL ongoing	Rectus gyrus	left	-12	35	-17	5.3	10	< .001
	Middle occipital gyrus	left	-18	-97	13	5.23	29	< .001
	Superior occipital gyrus	right	18	-97	16	4.62	1	< .001
PM ongoing > CTRL ongoing	Inferior parietal lobule	left	-30	-58	40	12.63	7486	< .001
	Supplementary motor area/superior medial frontal gyrus	left	-6	8	55	10.7	7762	< .001
PM ongoing < CTRL ongoing	Rectus gyrus	left	-12	35	-17	5.56	79	< .001
	Superior frontal gyrus	left	-18	41	52	5.11	9	< .001
	Anterior cingulum	right	6	35	-8	4.71	3	< .001

Supplementary Table 2. Significant regions for group x task interaction

Design	Contrast	Region	Side	MNI coordinates			Maximum *t*	No. of voxels	*P* value
				x	y	z			
Block	PP (PM-CTRL) < Control (PM-CTRL)	Superior temporal gyrus	left	-57	-10	-5	3.19	69	.001
		Hippocampus	right	27	-34	-8	3.13	28	.001
		Superiror temporal gyrus	right	63	-13	4	2.95	25	.002
Event-related	PP (PM-PM ongoing) > Control (PM-PM ongoing)	Supramarginal gyrus	left	-42	-49	34	3.52	74	< .001
		Paracentral lobule	left	-9	-22	70	3.45	38	< .001
	PP (PM ongoing-CTRL ongoing) < Control (PM ongoing-CTRL ongoing)	Superior parietal lobule	left	-18	-49	70	3.18	35	.001
		Hippocampus	right	27	-34	-5	3.1	26	.001

Note.- PP, postpartum group.

Supplementary Table 3. Significant group x task interaction regions for PPI analysis

Contrast	Region	Side	MNI coordinates			Maximum t	No. of voxels	*P* value
			x	y	z			
PP (PM-PM ongoing) < Control (PM-PM ongoing)	Superior occipital gyrus	left	-36	-97	16	4.47	369	< .001
	Superior medial frontal gyrus		0	59	-11	4.41	582	< .001
	Superior temporal gyrus	right	45	-64	25	4.02	172	< .001
	Cerebellum	left	-12	-73	-35	3.52	115	< .001
	Precuneus	right	9	-52	16	3.50	120	< .001
	Hippocampus	right	21	-22	-8	3.41	56	< .001
	Inferior parietal lobule	right	30	-40	52	3.40	96	< .001
	Fusiform gyrus	right	36	-46	-14	3.32	28	.001
	Cerebellum	left	-48	-61	-47	3.30	34	.001
	Cerebellum	left	-18	-79	-17	3.23	78	.001
	Middle occipital gyrus	right	27	-88	34	3.07	45	.001
PP (PM-CTRL ongoing) < Control (PM-CTRL ongoing)	Middle occipital gyrus	left	-48	-85	16	3.99	472	< .001
	Middle temporal pole	left	-30	5	-38	3.77	51	< .001
	Middle frontal gyrus	right	48	-7	58	3.54	40	< .001
	Vernis		0	-58	-50	3.39	40	< .001
	Lingual gyrus	right	15	-58	-2	3.29	77	.001
	Inferior temporal gyrus	left	-51	-1	-32	3.17	30	.001
	Superior frontal gyrus	right	24	-13	67	3.04	27	.001
	Middle temporal gyrus	right	51	-67	16	2.98	29	.002
PP (PM ongoing-CTRL ongoing) > Control (PM ongoing-CTRL ongoing)	Rectus gyrus	left	-3	53	-23	3.33	42	.001

Note.- PP, postpartum group.

Supplementary Table 4. Effect of predictors (serum level of estradiol, BDI score, and number of nocturnal awakenings) on PM accuracy through a mediator (FC_PM>PM ongoing) after controlling for age and duration of education.

	FC_PM>PM ongoing			PM accuracy		
	ß	SE	*P* value	ß	SE	*P* value
Estradiol (pg/mL)	.006	.002	.004	.001	.001	.444
BDI score	.048	.040	.228	.035	.021	.094
Number of awakenings	-.147	.213	.490	-.429	.110	< .001
FC_PM>PM ongoing				.191	.081	.018

Note.- ß, beta coefficient, FC_PM>PM ongoing, functional connectivity between the right hippocampus and retrieval-related ventral fronto-parietal network in PM trials compared to PM ongoing trials; SE, standard error
